# Associations of Dragonflies (Odonata) to Habitat Variables within the Maltese Islands: A Spatio-Temporal Approach

**DOI:** 10.1673/031.012.8701

**Published:** 2012-07-25

**Authors:** Mario V. Balzan

**Affiliations:** ^1^ Institute of Earth Systems, University of Malta, Msida, Malta; ^2^ Current address: Institute of Life Sciences, Scuola Superiore Sant'Anna, Piazza Martiri della Libertà, Pisa, Pl, Italy

**Keywords:** agriculture, biological indication, biodiversity assessment, community dynamics, insect conservation, landscape, macrophytes

## Abstract

Relatively little information is available on environmental associations and the conservation of Odonata in the Maltese Islands. Aquatic habitats are normally spatio-temporally restricted, often located within predominantly rural landscapes, and are thereby susceptible to farmland water management practices, which may create additional pressure on water resources. This study investigates how odonate assemblage structure and diversity are associated with habitat variables of local breeding habitats and the surrounding agricultural landscapes. Standardized survey methodology for adult Odonata involved periodical counts over selected water-bodies (valley systems, semi-natural ponds, constructed agricultural reservoirs). Habitat variables relating to the type of water body, the floristic and physiognomic characteristics of vegetation, and the composition of the surrounding landscape, were studied and analyzed through a multivariate approach. Overall, odonate diversity was associated with a range of factors across multiple spatial scales, and was found to vary with time. Lentic water-bodies are probably of high conservation value, given that larval stages were mainly associated with this habitat category, and that all species were recorded in the adult stage in this habitat type. Comparatively, lentic and lotic seminatural waterbodies were more diverse than agricultural reservoirs and brackish habitats. Overall, different odonate groups were associated with different vegetation life-forms and height categories. The presence of the great reed, *Arundo donax* L., an invasive alien species that forms dense stands along several water-bodies within the Islands, seems to influence the abundance and/or occurrence of a number of species. At the landscape scale, roads and other ecologically disturbed ground, surface water-bodies, and landscape diversity were associated with particular components of the odonate assemblages. Findings from this study have several implications for the use of Odonata as biological indicators, and for current trends with respect to odonate diversity conservation within the Maltese Islands.

## Introduction

Dragonflies and damselflies are a flagship group, and an important component of aquatic ecosystems, in which they can often be top predators. Their sensitivity to environmental conditions makes odonates excellent biological indicators of environmental conditions (Brown 1991; Clark & Samways 1996; Samways, et al. 2010). The ecological requirements of adults, which are associated with aquatic habitats and terrestrial landscapes, their selectivity of oviposition sites, and the influence of aquatic processes on the larval phase, which leads to their persistence in waterbodies, give rise to the familiar associations between each species and its characteristic habitat (Corbet 1993, 1999), making Odonata susceptible to specific types of habitat changes (Corbet 1993; Clark and Samways 1996; Schindler, Fesl, and Chovanec 2003; Foote & Rice Hornung 2005). The association of Odonata with their habitats, in addition to other characteristics of this taxon, which include their functional importance within ecosystems, and their association with other species and resources, makes surveys of odonate communities an important tool for characterizing and assessing the land-water interface through their function as indicators of ecosystem quality. The adults are conspicuous, easy to record, taxonomically well studied, and susceptible to habitat changes induced by human activities, all characteristics desirable of bioindicator groups (Brown 1991). Surprisingly, however, very little is known about the autoecological factors limiting species distributions to particular habitats (McPeek 2008), which may include sites and resources for larval development, emergence, adult foraging, mate seeking, oviposition, and nocturnal roosting.

Since Odonata inhabit both aquatic and terrestrial environments, they may better reflect environmental variation at different scales, ranging from the characteristics of the aquatic habitat, including the characterizing vegetation that plays an important role throughout the lifecycle of Odonata, to the surrounding terrestrial landscape that provides resources and conditions necessary for the persistence of the adult stages, than other species with obligatory freshwater stages. Nonetheless, despite being widely touted for their bioindication properties, only limited quantitative data on habitat relations of Odonata are available. Furthermore, while a lot of ecological work has led to an advanced knowledge of the ecological requirements of a large number of Odonata species, existing autoecological literature has been mainly discussed qualitatively, and statistical approaches remain rare (Schindler et al. 2003; Hofmann and Mason 2005). The odonate fauna of the Maltese archipelago is not an exception in this regard. Existing literature forms a series of faunal lists, each providing an updated account through the subsequent addition of descriptive observations and new records. Such lists are often made up of opportunistic data recording the presence of species, which may suffer from a number of biases, such as variation in observation effort over time (Kéry and Plattner 2007; van Strien et al. 2010). Consequently, little information is available on neither dragonfly population dynamics, nor the ecological needs of larval and adult stages within the highly spatiotemporally restricted waterbodies of the Islands.

The aim of this study was to investigate how odonate assemblage structure and diversity are influenced by changes in habitat variables associated with the aquatic environment and the surrounding agricultural landscapes. All study sites were located in predominantly agricultural landscapes of the two main islands of the Maltese archipelago, Malta and Gozo. This paper presents a multi-scale approach, which considers habitat variables ranging from the type of water body, to vegetation characteristics and landscape composition, in order to investigate the odonate - habitat relations within the Maltese Islands. Dragonfly communities are increasingly threatened with habitat loss and degradation within the Mediterranean Region from factors such as water pollution from agricultural practices (Riservato et al. 2009). Within the semi-arid climate of the Mediterranean, water resources may also be a key issue.

## Materials and Methods

### Study area

In the long cultural history of the Maltese archipelago, natural woodlands were transformed into mosaic-landscapes of agricultural and semi-natural habitats, making agriculture today the predominant land use, occupying around 46.8% of the 316km^2^ that compromise the land area of the archipelago (NSO 2006). Surveyed farmland and seminatural water-bodies within agricultural landscapes were classified according to the physical characteristics of the water-body, and the availability of water resources (Table 1).

The Maltese Islands have a typical Mediterranean climate, characterized by hot, dry summers, and mild, wet winters (Chetcuti et al. 1992), with average rainfall of 550 mm for the period 1900–2000, but with high seasonal and inter-annual variability (FAO 2006). Due to high demand for water resources, abstraction of groundwater, which is essential to sustain the relatively limited perennial surface water ecosystems, also exceeds aquifer recharge rate (FAO 2006). Moreover, agricultural water demand is projected to increase, and is significantly higher during the summer months (FAO 2006). The estimated amount of irrigated land amounts to 29% of the total utilized agricultural area (NSO 2010). Consequently, agricultural reservoirs have become an essential agricultural feature within the Maltese Islands. These water bodies, though typically small and lacking riparian vegetation, may potentially provide habitat for several Odonata species within the rural landscape. Over the years, several valley watercourses have been substantially modified since the agrarian boom, dating back to the early 1980s, to ensure higher rainwater tapping and storage, thus reducing natural surface water flow and persistence throughout the drier months. Water-use pressures, which are likely to be exacerbated by global warming and increased agricultural water demand, together with pollution, habitat degradation, and the recent influx of several predatory alien species (e.g. *Gambusia* spp., *Rana bedriagae* (Camerano, 1882), and *Trachemys scripta elegans* (Wied-Neuwied, 1839)) in particular waterbodies (Sciberras & Schembri 2006; Balzan 2008a) is suggested to provide a high level of threat to Odonata within the Maltese Islands. The odonate fauna of Malta has been recently reviewed by Ebejer et al. (2008). More recently, another species, *Orthetrum nitidenerve* (Selys, 1841), was recorded (Sciberras et al. 2010), making 16 total species ever recorded in scientific literature within the archipelago.

### Monitoring of adult dragonflies

Water bodies were surveyed on ten occasions from mid-April to mid-July 2008 in order to take into consideration seasonal changes in Odonata fauna, as well as the temporal availability of water. Standardized survey methodology for adult male Odonata involved timed, 30-minute direct counts, made while walking through riparian habitats, of male individuals flying over water and emergent vegetation, or within 2 meters of the waters' edge. Survey biases from diurnal movement of dragonflies were avoided by deliberately varying the time of day at which surveys were conducted at each site and sampling period. Within the life-cycle of Odonata, subsequent to maturation in terrestrial habitats away from stream sites, males arrive at the breeding habitat where they set up territories, actively defend their territories from other intruding males, and court females. Males arrive at the aquatic habitat during the morning, and remain there until late-day departure to roosting sites. Indeed, density within breeding grounds has been shown to remain fairly constant due to territorial behaviour, and direct counts over water-bodies are considered a conservative measurement of odonate abundance (Moore 1953; Conrad et al. 1999). Similar timed counts were conducted over farmland and semi-natural water bodies. Where necessary, a hand-net was used to catch Odonata for identification, after which they were released. Species identification was based on d'Aguilar et al. (1986) and Askew (2004).

### Larval Sampling

While adult choice of habitat probably plays a proximate role in setting the environmental limits, species distributions, these are probably ultimately set by processes acting on the aquatic larval phase (McPeek 2008). A survey of Odonata larvae was carried out from January-March, of the same year; this is the period when adult Odonata are not usually observed on the wing. During this time-frame, many resident taxa are present in the larval stage, no migratory dragonflies are normally present, and the wetter conditions during this period provide ample surface-water habitat for larval Odonata. A standardized sampling methodology for collecting larval stages consisted of five 1m sweeps, using a rectangular dredging net (200 × 450 mm) with a mesh size of 1 mm, on two occasions for each site. Given the importance of aquatic macrophytes, which provide oviposition sites for adults, and concealment for larval stages of several dragonfly taxa (Corbet 1962 1999), each visit to the site consisted of three standardized 1m sweeps between the beds of vegetation, and another two sweeps carried out within the water body over open sediment. Collected mud was rinsed with tap water, and filtered using a set of sieves with decreasing mesh size (up to 0.5 mm). All samples were sorted within 24 hours after collection, and collected specimens were preserved in 70% ethanol solution. A dissecting stereomicroscope (Leica GZ6, 6.7–40× magnification) was used for subsequent larval identification, which was based on Askew (2004).

### Survey of riparian vegetation

The pervasive role played by vegetation in the odonate life cycle makes it, a priori, likely that macrophytes are prominent among cues used for habitat selection (Corbet 1999). Literature suggests that Odonata assemblages are influenced by the presence of aquatic plants (Clark & Samways 1996; Stewart & Samways 1998; Schindler et al. 2003; Samways & Taylor 2004; Hofmann & Mason 2005), with the structure and the ‘architecture’ of the plants, or the communities they form, likely to be important for adult habitat selection (Corbet 1999).

Vegetation assessment of the sites consisted of two 10 m belt transects of 1 m width over riparian vegetation at the land-water interface of the survey sites for odonate diversity, lotic water bodies, or the long side of lentic water bodies. Meanwhile, four 5 m by 1 m transects were laid perpendicular to these, with the aim to provide an indication of how vegetation changed with increasing distance from the water bodies. Within these transects, plant abundance, distribution data, and physiognomic characteristics, in terms of vertical stratification and characteristic life form, were obtained from a series of contiguous quadrats laid down along a transect. A square (1 m × 1 m) quadrat was used for this study. The quadrat was subdivided into a number of sectors of equal area in order to increase accuracy of recording when measuring abundance (Kent & Coker 1995). The abundance of each species within each quadrat was assessed by counting the number of sectors within which the species was represented, either in whole or in part. This figure was expressed as a proportion of the total number of sectors in the quadrat.

For each quadrat, vertical stratification (the arrangement of phytomass into vertical layers) was measured by counting the number of interceptions per species at each height class at the centre of the quadrat. A 20vmm diameter, 4 m height wooden pole was divided into 7 height classes (0–0.20, 0.21– 0.50, 0.51–10, 1.01–1.50, 1.51–2.00, 2.01–2.50, 2.51–3.25, 3.25–4.00, > 4m). The height of vegetation was measured from the ground to the height of the highest vegetation shoot, with vegetation > 4m considered as canopy cover. In addition to vertical stratification, the physiognomy of the vegetation may also be profoundly affected by the life form of dominant vegetation (Kuchler 1988), that is the presence of common morphological features that are usually assumed to be an adjustment to important environmetnal factors (Mueller-Dombois and Ellenberg 1974; Zonneveld 1988). The percentage cover of the various life forms present within the site (graminoids, forbs, subshrubs, shrubs and trees), as well as details relating to species composition, were collected for each quadrat.

### Quantifying the landscape pattern

The terrestrial landscape surrounding the aquatic breeding habitat provides several resources and conditions that are critical for the species, making the adjacent landscape probably as important as the aquatic habitat itself (Corbet 1999). The landscape surrounding semi-natural and freshwater bodies (n = 6) was surveyed through the use of orthophotos, taken in 2004, and having a resolution of 1 pixel: 15 cm. Fiddien and Qlejgħa form part of the same valley system and given their physical proximity only the former site was considered. Different land use and land cover categories (Table 2) were identified and mapped as polygons. Digitized data had the same level of resolution as the ortophotos. Landscape metrics were derived from Patch Analyst 4, an ArcGIS extension which enables spatial analysis of landscape patches (Rempel 2008) At the landscape level, the Shannon diversity index (SDI) was used to quantify the diversity of the agricultural landscapes. In addition, the mean perimeterarea ratio (MPAR), and the number of patches for the various landscapes under study, were also obtained. At the class level, the number of patches (NP), the proportion of the landscape (P_i_), the mean patch size (MPS), and the MPAR were calculated.

### Data Analysis

**General patterns for Odonata.** Because the number of sampling occasions for the sites varied, as a result of the differential availability of water at the different study sites, the data was standardized by averaging the total number of recorded individuals for each species on ten visits for each water body. Renyi diversity profiles (Tóthmérész 1995), which provide information on richness and evenness of study sites, were used to compare the diversity of odonates between semi-natural sites and habitat types (Kindt and Coe 2005; Oksanen et al. 2010). The major advantage of Rényi diversity profiles is that sites can easily be ordered from high to low diversity. Subsequently, the Shannon diversity index (H) was used to provide a measure of relative diversity for Odonata and larvae, as well as vegetation diversity. The diversity values for odonate larvae were not normally distributed (Shapiro-Wilk test, W = 0.7551, p = 0.006), and subsequent analysis with these values was non-parametric.

**Multiscale habitat variation and Odonata.** In order to analyze the association of different species to the environmental variables at the scales studied, a principal component analysis was carried out using the vegan package in R (Oksanen et al. 2010). PCA is a multivariate technique based on linear assumptions, which is preferable to use when the species has low beta diversity (Lepš and Smilauer 2003), as was the case with the data in this study. To aid interpretation, the environmental variables were fitted onto the PCA ordination plot using the Vegan ‘envfit’ function (Oksanen et al. 2010). On an ordination plot, this function fits a centroid of levels of a class variable, and calculates an R^2^ value as a measure of separation among the different levels of that variable. Additionally, a significance value for the R^2^ was calculated using 1000 random permutations of the category levels. Explanatory variables identified to have a p-value < were subsequently correlated with PC1 and PC2 site scores through a Pearson's correlation (R Foundation for Statistical Computing 2010).

**Habitat and time as determinants of odonate assemblage similarity.** Partitioning of factors across spatio-temporal scales that influence population and community structure is particularly important when studying complex ecological systems. Moreover, such tools are especially useful in testing hypotheses of experimental designs involving several factors, which are often characteristic of several multivariate studies. Traditional multivariate methods, such as multivariate analysis of variation (MANOVA), rely on the assumption that data conforms to a multivariate normal distribution, a condition that is not normally met by abundance data, since these are usually highly aggregated or skewed. The community data consisting of species records from six sites in the present study were significantly non-conformant to multivariate normality (W = 0.3159, p = 5.414e-07) when tested using the Shapiro-Wilk Multivariate Normality Test (Slawomir 2009). Choice of study sites for this analysis was limited to semi-natural water bodies with a permanent water supply throughout the duration of the monitoring programme. Permutational multivariate analysis of variance (perMANOVA) was used, through a nested design based on site and sampling occasion, to investigate the effect of habitat type (lentic and lotic) and time on adult Odonata assemblages within the study sites, the null hypothesis being that Odonata community assemblages in lotic and lentic water bodies are ecologically similar through time. PerMANOVA is a function for the analysis and partitioning of sums of squares using semimetric and metric distances (Anderson 2001; McArdle and Anderson 2001). PerMANOVA analyses were performed in R with the ‘adonis’ function in the vegan package using the Bray-Curtis distance measure and 1000 permutations. To further evaluate the influence of time on individual species abundance, a generalized linear model (GLM) using a quasi-Poisson distribution (log-link function) was performed on the count data of the two sites (R Foundation for Statistical Computing 2010). The quasi-Poisson model uses the mean regression and variance functions from the Poisson GLM, but unlike the latter, it leaves the dispersion paramenter unrestriced. A Wald test was subsequently used to provide an overall fit of the model (Zeileis and Hothorn 2002).

## Results

### Adult Dragonflies Monitoring

**Figure 1.**
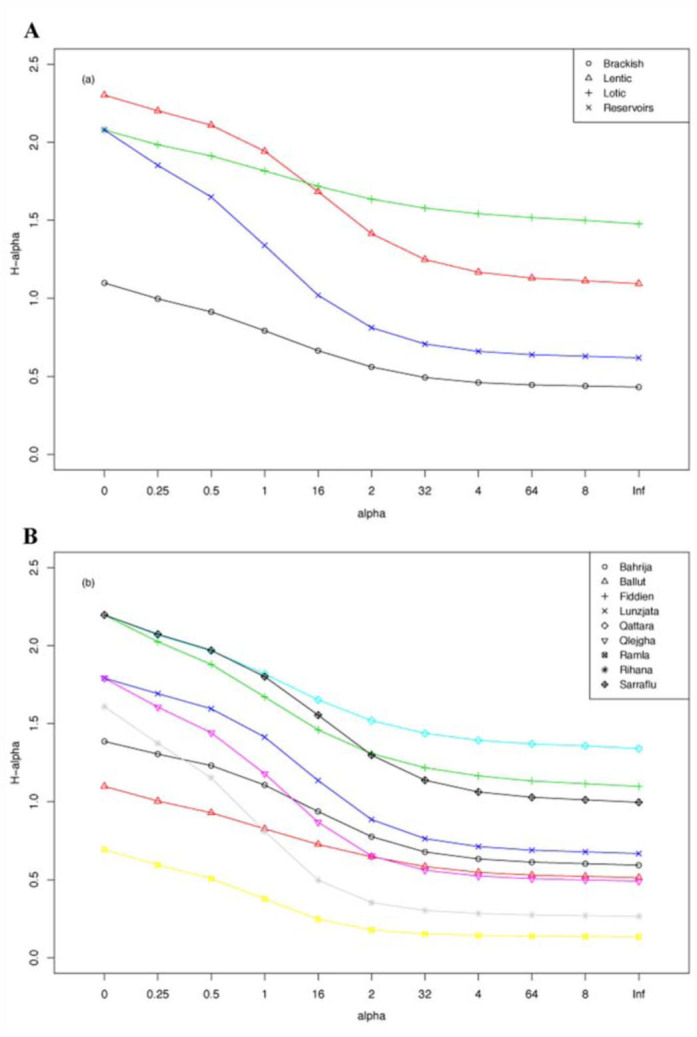
Renyi profiles comparing adult Odonata diversity of (a) different types of habitats, and (b) for the semi-natural sites of the study area. High quality figures are available online.

Temporal availability of water within the selected aquatic habitats varied. In one of the water bodies (Wied Hesri), water presence was limited to the wetter months, and thus the habitat was unable to host any adult odonates. Consequently, it was omitted from adult Odonata monitoring. In total, 643 adult odonate individuals belonging to nine species were recorded. Since water persistence varied with site, species abundance data was standardized for each water-body (Table 3). These standardized data were used in subsequent data analyses, unless otherwise stated. The two sites characterised by higher salinity levels, Ballut (Marsaxlokk) marshland and Ramla 1-Hamra (Gozo), showed the lowest level of species diversity. A comparison of the Renyi profiles (Figure 1a) produced for all four types of habitats revealed that lentic and lotic semi-natural waterbodies were normally more diverse than agricultural reservoirs and semi-natural habitats with higher salinity levels. For agricultural reservoirs, two species, *Anax imperator* (Leach 1815) and *Crocothemis erythraea* (Brullé 1832), were the most abundant, with the other species only recorded on single occasions. A comparison of Renyi profiles for lotic water-bodies with a perennial and ephemeral water availability did not show any difference in Odonata diversity (Figure 1b). A paired t-test, to compare the abundances of recorded odonates in both types of watercourses, suggested that they were not significantly different (t = -0.560, p = 0.591).

### Larval Sampling

Larval sampling methodology only yielded odonates from four sites: Għajn Rihana, IlFiddien, Il-Qattara, and Ta' Sarraflu. No larval Odonata were recorded along the Baħrija, Qlejgha, Hesri, and Lunzjata watercourses. Similarly, larval Odonata sampling in brackish waters of Ballut marshland (Marsaxlokk) and Ramla 1-Ħamra watercourse yielded no results. Higher larval diversity was not significantly associated either with higher diversity, as measured with the Shannon's diversity index (H), of the same species (Spearman's rho = 0.55, p = 0.12), or with higher Odonata assemblage species diversity for study sites (Spearman's rho = 0.58, p = 0.10).

**Figure 2.**
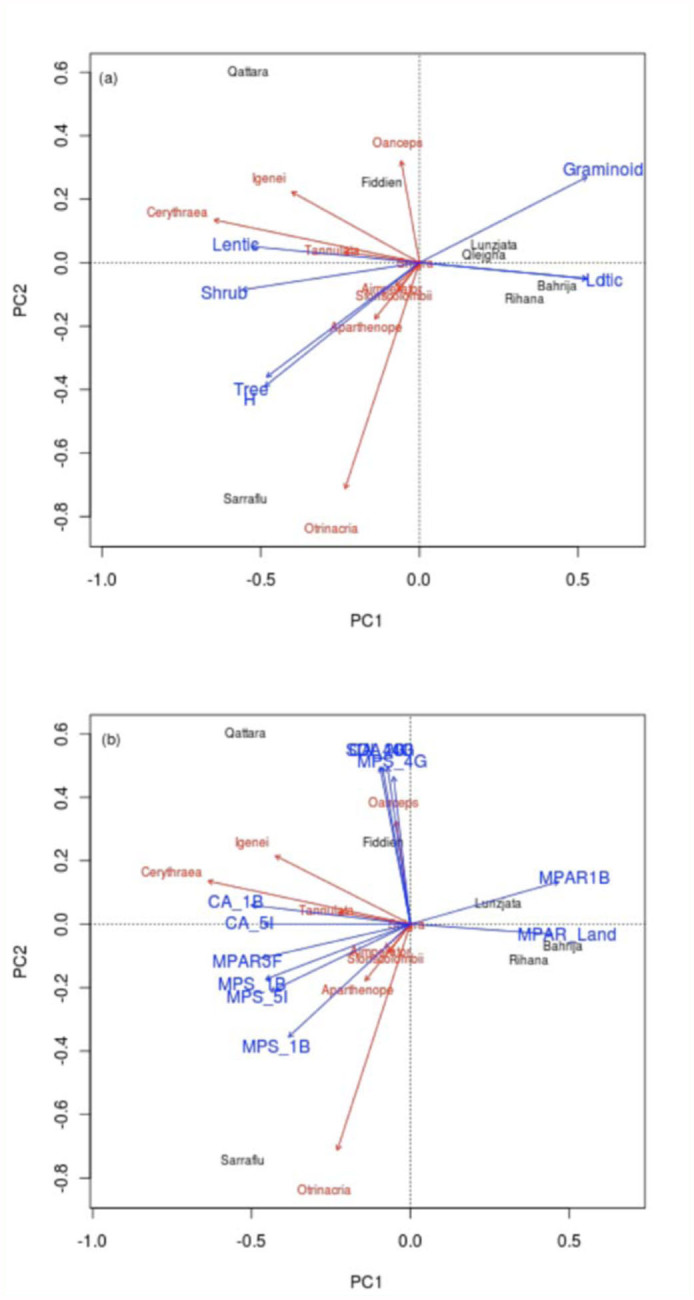
Ordination graph illustrating the outcome of an indirect gradient analysis (PCA), followed by environmental fitting of explanatory variables (only vectors with p-value ≤ 0.1 are included) for (a) vegetation and (b) landscape variables. High quality figures are available online.

### Riparian vegetation survey

A total of 49 species were recorded during the vegetation survey performed within the nine semi-natural waterbodies investigated. The most abundant species was the giant reed (*Arundo donax* L.), an archaeophyte that has become highly invasive, with its dense monospecific stands having colonized various waterbodies within the archipelago. The graminoid life form and canopy cover classes of 3.25–4 m and > 4 m categories were globally the most abundant groups within study sites. Odonata and vegetation diversity (Table 4), measured through the Shannon diversity index, were found to be significantly correlated (R = 0.795, p = 0.01).

### Multiscale habitat variation and Odonata !

The PCA results identified clear patterns in species composition, with the first two components explaining 78.9% and 13.2% of the variation respectively. PC1 was negatively weighed on the population data of *C. erythraea* and *Ischnura genei* (Rambur 1842), while PC2 was strongly related to the presence of *Orthetrum trinacria* (Selys 1841), and to a smaller extent inversely to that of *Orthetrum coerulescens anceps* (Schneider 1845) (Figure 2). Lentic sites were on one end of the PC1, while brackish and lotic sites were at the other. Most of the species were more strongly correlated with the lentic sites, indicating the importance of the latter for the conservation of dragonfly species of the Maltese Islands. Variation was in turn correlated with several environmental variables. Nonetheless, some of the variables show some collinearity, as may be clearly observed from Figure 2a. For example, total abundance of graminoid species, of which abundance of *A. donax* accounted for 55.8%, and individuals in height class I (> 4m) show some correlation with lotic sites. Similarly, tree life form abundance and the height class H (3.25–4m) were associated with the Ta' Sarraflu freshwater pond.

Subsequent correlation identified several variables at multiple scales which could influence the distribution of Odonata within the study sites (Table 5). A high R value for a given explanatory variable may be because the variable exerts an important control on Odonata assemblages, or the variable may be correlated with another variable that influences odonates. At the water-body scale, *C erythraea* and *I. genei* were found to be significantly negatively correlated to the abundance of graminoids and *A. donax*, as well as canopy cover > 4m. In contrast, they showed a positive correlation with shrub and tree life-form abundances. At the landscape level, these species were positively correlated with MPS (200 m) and class area (r = 500m), and negatively correlated to the MPAR (r500m), of ecologically disturbed class cover. They were also related positively to MPAR of coastal areas, and the class area of marine habitat, both of which are probably the result of the main habitats for these species within the studied sites being located in the vicinity of the coast. *O. trinacria*, unlike *O. coerulescens anceps*, was found to be correlated to low landscape diversity, and a lack of semi-natural freshwater habitats class area, a category which mainly consists of valleys and watercourses within the surrounding landscapes at both scales (r = 200, 500 m).

**Figure 3.**
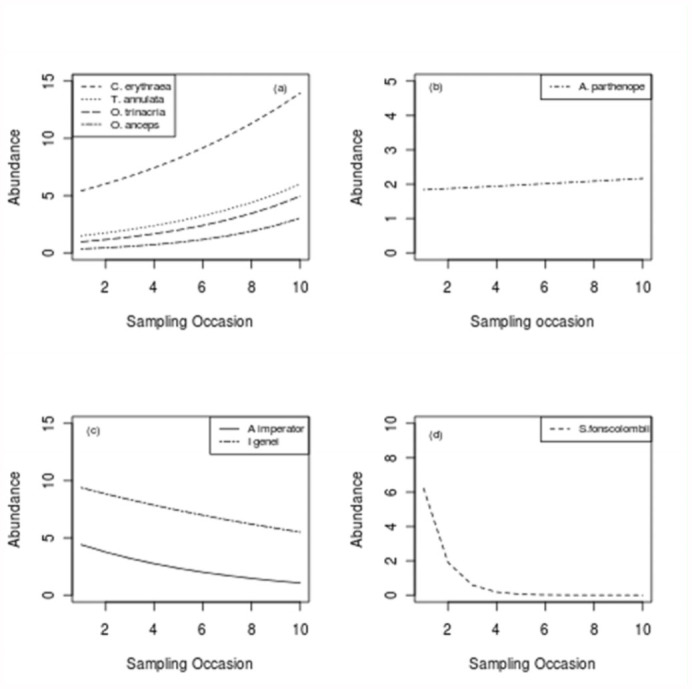
Odonata — time relationships according to type of functional response. High quality figures are available online.

### Habitat and time as determinants of odonate assemblage similarity

The perMANOVA results suggest that odonate assemblage similarity is significantly influenced by habitat type (p = 0.0004) and sampling period (p = 0.02), thus rejecting the null hypothesis. The interaction between habitat type and sampling occasion was found to be not significant (Table 6). A comparison of odonata counts with sampling period suggests that different species adopt different life-history strategies.

The GLM for species abundances data at these sites suggests that time significantly influences the abundance of adults at the breeding site for several species (Table 7). Since *S. nigra* was sporadically recorded in low abundance, abundance data for this species were not considered for this part of the data analysis. Given the relative importance of habitat type on dragonfly distributions, the total Odonata counts from Il-Qattara and Ta’ Sarraflu were used in order to investigate assemblage structure through time (Figure 3). A paired t-test suggested that dragonfly counts, taken on the same days, from both sites were not significantly different (t = 0.10, P = 0.92). This result indicates the importance of including repeated measures in similar studies, including ones aimed at assessing conservation value of habitats, and the use of Odonata as biological indicators.

## Discussion

Of the 16 total odonate species noted for the Maltese Islands, only nine taxa were recorded within the surveyed water-bodies. Two species, *O. trinacria* and *Trithemis annulata* (Palisot de Beauvois 1807), that have only been recently recorded (Ebejer et al. 2008) probably have established populations (Balzan 2008b). These two species were found associated with different types of aquatic habitats. Conversely, the lack of records of species, such as *Orthetrum cancellatum* (L. 1758) and *Sympetrum striolatum* (Charpentier 1840), previously considered as common within the Maltese Islands, and other less abundant species such as *Orthetrum brunneum* (Fonscolombe 1837), may suggest that populations of Odonata on the Islands are on the decline. Similar observations have been reported for several European (Sahlén et al. 2004) and Mediterranean (Riservato et al. 2009) species.

Habitat-odonate relations identified in this study have some important implications for dragonfly monitoring, and the use of Odonata as bioindicators within the Maltese Islands. A primary environmental feature determining the habitat distributions of Odonata is water-body type. Water-bodies characterized by brackish conditions showed relatively low adult Odonata diversity, and no larvae were collected from the habitats surveyed throughout the study. Similarly, farmland reservoirs showed considerably lower odonate diversity when compared to semi-natural water-bodies. In contrast, the importance of artificially created habitats in maintaining populations of several dragonfly species, and their positive contribution to the conservation of Odonata, has been documented elsewhere (Clark and Samways 1996; Samways 1989).

Nevertheless, the reservoirs surveyed here differ from man-made habitats described in the latter studies, and typically have a small surface-area, lack macrophytes, and are likely to host predatory fish populations (Balzan 2008a).

While many odonate taxa require specific habitat features (Corbet 1999; McPeek 2008), most of the species recorded during adult counts within the study area were found in both lotic and lentic water-bodies. Only two species, *S. nigra* and *O. trinacria*, were entirely confined to lentic habitats. This may suggest that the Odonata fauna of Malta is mainly composed of euryoecious species that are able to utilize different aquatic habitats along a flowing-standing water continuum of habitat types. This contrasts with other studies investigating the influence of habitat characteristics on Odonata assemblages that have identified particular dragonfly associations to specific habitat types (Clark and Samways 1996; Samways 1996; Schindler et al. 2003). Habitat associations of the recorded species obtained by PCA suggest that even though most of the odonate species recorded were recorded in lotic water-bodies, nearly all species were more strongly associated with the lentic habitats. Records of larval Odonata were mainly confined to lentic water-bodies, confirming observations made in other studies that adult sightings may not always predict larval distribution (Painter 1998; Corbet 1999), and indicating that standing water-bodies within the study area may be particularly important for dragonfly conservation. Similar observations have been made elsewhere, where it has be suggested that in rapidly changing lotic environments, which tend to dry up during the dry season and are often fast-flowing and scouring during the wet season, larval survival following oviposition is relatively lower (Samways 1989; Hofmann and Mason 2005). Contrastingly, lentic water-bodies within the Maltese Islands provide a distinct type of habitat, characterized by perennial water availability, and relatively higher vegetation diversity (Table 4).

The importance of macrophytes in influencing Odonata assemblages has been identified for several ecosystems (Clark and Samways 1996; Samways 1996; Stewart and Samways 1998; Painter 1999; Schindler et al. 2003; Samways and Taylor 2004; Carchini, Solimini, Ruggiero 2005; Hofmann and Mason 2005). Correlations investigating the influence of plant species on Odonata assemblages within this study appear to confirm the hypothesis made by Corbet (1999) that structure and appearance of plant communities, rather than individual plant species, are likely to serve as cues for habitat recognition. For example, odonates of Malta within lotic habitats may be particularly threatened by the presence of invasive *A. donax* reed stands, which were significantly correlated to PC1 for Odonata abundance data, indicating its negative influence on species such as *C. erythraea* and *I. genei*, the latter being an endemic of Mediterranean Islands (Corsica, Sardinia, Sicily, Maltese Islands, Capraia, Elba and Giglio). Conversely, these species were correlated with shrub and tree abundances. Odonata species have been reported to be particularly threatened by invasive alien plant species along watercourses (Samways and Taylor 2004), which alter sunlight versus shade regimes along aquatic systems (Samways 2005). Moreover, in a 27 year study on dragonfly communities of small ponds, Moore (1991) observed a decline in the population of adults that coincided with extensive growth of reed. The dense stands impaired dragonfly flight, and reduced the growth of submerged or floating macrophytes. In a separate study investigating the management of ditches, it was observed that densely reeded sites were rarely used by territorial males and ovipositing females, which favored ditches with little shading from bankside vegetation (Painter 1998).

In light of the rapid changes characterizing agricultural landscapes, an important issue is the scale at which the habitat is measured. The habitat has been defined as the collection of resources and conditions required by, and accessible to, individuals of a species at a location (Dennis et al. 2003, 2007). Because the habitat is necessarily the location where an organism lives out its life-cycle, a suitable habitat must meet the ecological needs of all life-stages. The terrestrial landscape is probably as important as the aquatic habitat (Corbet 1999), as it provides several conditions and resources that are required by the adult phase. Previous studies have indicated that responses in abundance and/or occurrence to local waterbody and landscape characteristics differ among species (Conrad et al. 1999) and life-history groups (Samways 1996; Krawchuk and Taylor 2003; Kadoya et al. 2008). PCA results suggest that the presence of *C. erythraea* and *I. genei* were related to disturbed areas, coastal land cover, and marine habitats, all of which characterize the landscapes of Il-Qattara and Ta' Sarraflu water-bodies in which these two species were the most abundant. *O. trinacria*, which has been recently recorded in the Maltese Islands (Ebejer et al. 2008), was recorded in all lentic water-bodies, but with a relatively higher abundance at one site that is characterized by a low landscape diversity and a lack of valley systems within the landscape. Conversely, *O. coerulescens anceps* appeared to be related to relatively heterogeneous landscapes, and the presence of associated lotic water bodies.

These contrasting associations for the two *Orthetrum* spp. may be a result of the different habitat preferences of these species, with *O. coerulescens anceps* normally associated with small ponds and streams with a gentle current, while *O. trinacria* is associated with large bodies of standing water (Askew 2004). The availability of suitable habitat that is likely to maintain source populations (*sensu* Pulliam 1988), such as gently flowing streams, is likely to determine the distribution of *O. coerulescens anceps* within these lanscapes. The multiscale approach adopted within this study permits the identification of unique habitat characteristics for different sites, and the influence these could potentially have on odonate community structure.

Odonate population dynamics were also related to the sampling period (Figure 3), a factor which is clearly related to the reproductive ecology of the surveyed species. *Sympetrum fonscolombii* (Selys 1840) was recorded only in the spring period, with their population subsequently declining to nil. Populations of *A. imperator* and *I. genei* reached a higher population count early in spring, and their population decreased with time for the rest of the flight period. Contrastingly, count data for *C. erythraea, O. Trinacria*, and *O. coerulescens* anceps increased with time, reaching the highest values near to the yearly temperature peak. This model may not be directly related to the life-cycle of Odonata, since other factors such as migration, intra and inter-specific competition, and water resources availability may play an important role in determining the occurrence and/or abundance of adult Odonata at the breeding habitat. Nevertheless, these results provide an indication of the population dynamics of Odonata across a temporal period within these habitats, which inherently has important implications for the characterization of Odonata-habitat associations, and in their application as biological indicators within the study area.

The identification of odonate-habitat associations is an essential tool for characterizing the response of dragonflies to changes in the environment. This study identified several variables across different spatio-temporal scales that may influence the occurrence and abundance of Odonata in aquatic habitats. The scales considered several factors, including physiochemical, vegetation characteristics of the water-bodies, and landscape composition, that influence odonate assemblages. Moreover, larval collection and repeated adult sampling at the breeding sites gave different results, indicating the importance of considering temporal variation in studies aimed at monitoring Odonata. All odonate species were recorded as adults over lentic water-bodies, and larvae were normally associated with these aquatic habitats, delineating the importance of lentic habitats in supporting odonate populations within the Maltese Islands. It may be concluded that the presence of adults along a water-body may not necessarily indicate the suitability of the habitat for larval stages, and several water-bodies may be colonized as a result of dispersal from parent populations. While the limited availability of water resources makes interpretation of the relative importance of measured environmental variables difficult to assess, results obtained from this study suggest that Odonata assemblage structure is influenced by factors across spatial and temporal scales, illustrating the need for further research on the autoecology of this taxon to consider these variables.

**Table 1.**
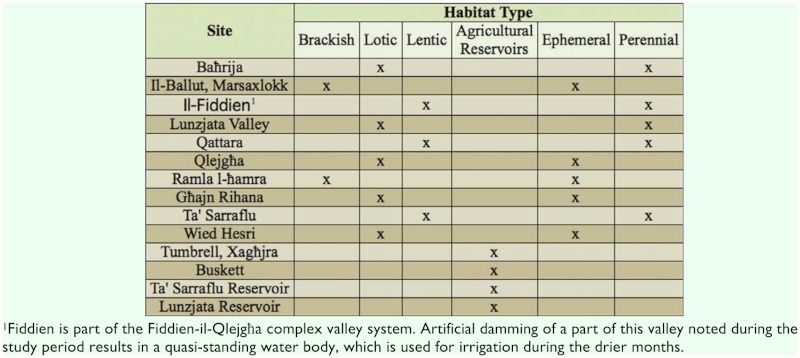
The types of water bodies and their distribution at the sites investigated during this study.

**Table 2.**
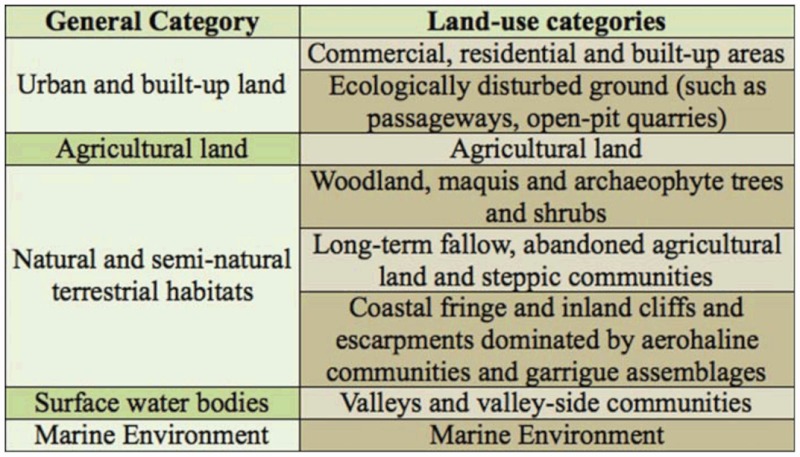
Land-use cover classification system used for the landscape analysis.

**Table 3.**
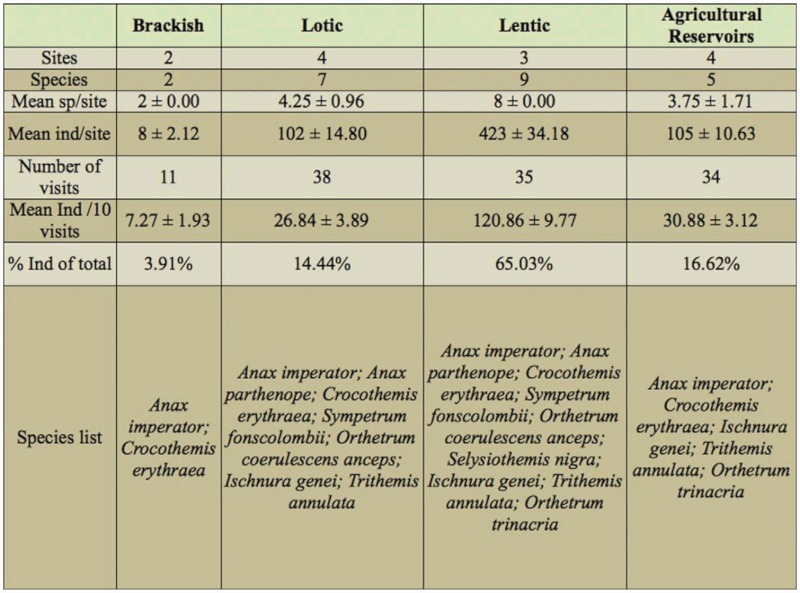
A comparison of the number of species and their abundance in the four different types of habitats.

**Table 4.**
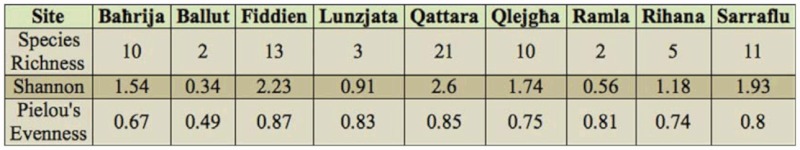
Vegetation data for the study sites.

**Table 5.**
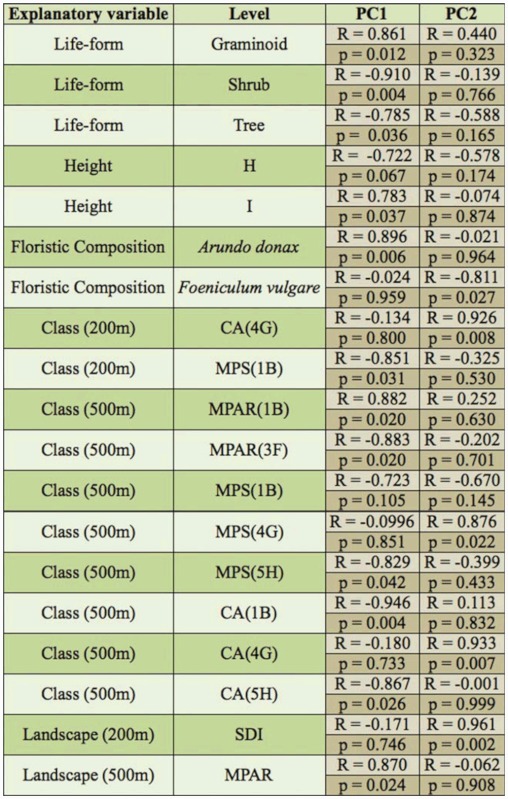
Correlation of PC1 and PC2 from a principal component analysis of Odonata abundance data with explanatory variables.

**Table 6.**
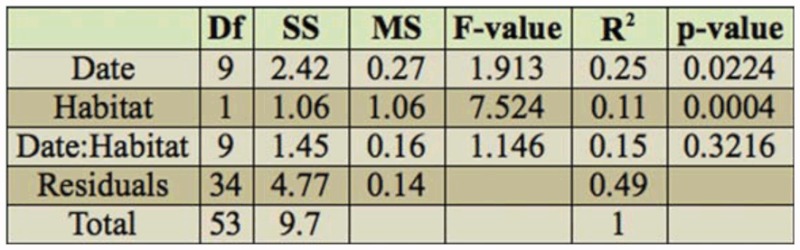
The influence of time (date of sampling occasion) and type of habitat (lotic and lentic) on Odonata assemblage similarity.

**Table 7.**
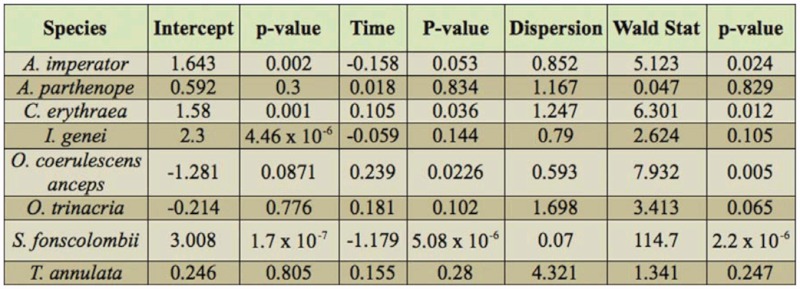
The outcome of a generalised linear model (quasi-Poisson, log-link function) of species abundance data with sampling period.
